# The Diagnostic and Immunotherapeutic Value of CD248 in Renal Cell Carcinoma

**DOI:** 10.3389/fonc.2021.644612

**Published:** 2021-03-12

**Authors:** Keying Zhang, Chao Xu, Shaojie Liu, Yao Jiang, Xiaolong Zhao, Shanjin Ma, Yu Li, Fa Yang, Yan Wang, Ping Meng, Changhong Shi, Donghui Han, Weihong Wen, Weijun Qin

**Affiliations:** ^1^Department of Urology, Xijing Hospital, Fourth Military Medical University, Xi'an, China; ^2^Department of Urology, Tangdu Hospital, Fourth Military Medical University, Xi'an, China; ^3^Department of Emergency, 987th Hospital of the Chinese People's Liberation Army, Baoji, China; ^4^Laboratory Animal Center, Fourth Military Medical University, Xi'an, China; ^5^Institute of Medical Research, Northwestern Polytechnical University, Xi'an, China

**Keywords:** renal cell carcinoma, CD248, immunotherapy, prognosis, molecular cancer signature

## Abstract

**Background:** Renal cell carcinoma (RCC) is the most common malignancy in the urinary system. Despite substantial improvements in available treatment options, the survival outcome of advanced RCC is unsatisfactory. Identifying novel biomarkers to assist in early diagnosis and to screen patients who are sensitive to immunotherapy would be beneficial. CD248 is a promising candidate that deserves to be investigated.

**Methods:** The Cancer Genome Atlas (TCGA) data set and clinical specimens were adopted to analyze the expression of CD248 between normal and tumor tissues. Univariate and multivariate Cox regression analyses were employed to identify independent prognostic factors and construct a CD248-based prognostic signature. The correlation among the present signature, tumor-infiltrating immune cells (TIICs), the tumor mutation burden (TMB), and immunomodulatory molecules was evaluated. The weighted gene co-expression network analysis (WGCNA), the enrichment analysis, and the miRNA correlation analysis were performed to explore the underlying mechanism of CD248 in the progression of RCC.

**Results:** The overexpression of CD248 in RCC was related to a poor prognosis, and a CD248-based prognostic signature could precisely stratify patients with RCC with different survival outcomes regardless of the training or testing cohort. The present signature could reflect the immunosuppressive landscape of RCC (i.e., increased infiltration of regulatory T cells and upregulated immune checkpoints), accompanied by deteriorated clinicopathologic indexes. The TMB and immunostimulatory molecules expression also increased with the risk score generated from the present signature. CD248 co-expressed gene sets were identified through the WGCNA algorithm, and several immunosuppressive Gene Ontology (GO) terms and Kyoto Encyclopedia of Genes and Genomes (KEGG) pathways were significantly enriched. The result of CD248-correlated miRNA further emphasized the importance of CD248 in RCC.

**Conclusion:** CD248 is a valuable biomarker to improve the diagnostic and therapeutic efficiency of RCC. The immunosuppressive effect of CD248 co-expressed genes may provide insight for the present study, and miRNA would help to reveal the mechanism of the expressive regulation of CD248.

## Introduction

Renal cell carcinoma (RCC) is the seventh most common neoplasm in the developed world and the most lethal malignancy in the urinary system (1). As reported, the morbidity of RCC has more than doubled in North America and Western Europe over the past half century and is predicted to rapidly increase in Latin America, Asia, and Africa in the coming decades (2). Actually, RCC is an insidious neoplasm with one-third of cases initially diagnosed as metastatic, whose survival rate is abysmally low. Despite treatment options for RCC have been revolutionized by targeted therapy, the 5-year survival rate of advanced/metastatic RCC is only 12% (3). Immunotherapy with immune checkpoint inhibitors to block PD1, CTLA4, and LAG3 is another promising method to promote the survival outcome of patients with RCC (4–6). However, the low response rate restricts its therapeutic efficacy (7). Hence, identifying novel biomarkers to facilitate the early diagnosis of patients who are asymptomatic and to assist clinicians for screening the ones who are sensitive to immunotherapy would be beneficial for the prognosis of RCC.

Tumor endothelial markers (TEMs) involved with tumor-specific angiogenesis play a crucial role in the development and progression of tumors, among which TEM1 (also known as endosialin or CD248) is specifically overexpressed in tumor-associated fibroblasts and pericytes residing in tumor blood vessels. It has been found that CD248 is an essential molecule associated with cell adhesion, migration, and stromal cell proliferation (8). Once CD248 is knocked out in mice, there was a striking reduction in the growth of the tumors, invasiveness, and metastasis after tumor transplantation, indicating that CD248-positive stroma would promote malignancy (9). Therefore, CD248-characterized tumor vasculature (10) and stroma (11) were regarded as promising targets for the therapy of tumors. However, whether CD248 can predict the prognosis of RCC and guide immunotherapy is largely unknown.

To explore the predictive value of CD248 in RCC, we conducted the present study. The Cancer Genome Atlas (TCGA) data set and clinical specimens were adopted to analyze the expression of CD248 between normal and tumor tissues. Then, we constructed a CD248-based prognostic signature by integrating multiple clinical variables, which acquired the promoted predictive accuracy. The correlation among the present signature, tumor-infiltrating immune cells (TIICs), the tumor mutation burden (TMB), and immunomodulatory molecules was also evaluated. Finally, the weighted gene co-expression network analysis (WGCNA) and enrichment analysis were performed to explore the underlying mechanism of CD248 in the progression of RCC.

## Materials and Methods

### Data Source and Preprocessing

Renal cell carcinoma data (895) and non-tumor data (128) were downloaded from the TCGA portal (https://portal.gdc.cancer.gov/). Transcriptomic data [RNA sequencing (RNA-Seq) Fragments Per Kilobase Million (FPKM)], miRNA isoform expression, and clinical information were integrated through ID numbers. The genes measured with multi-probes were replaced with their average *via* limma package (12). All data were processed and analyzed with R software (https://www.r-project.org/).

### The Differential Expression Analysis of CD248

Differentially expressed genes (DEGs) between tumor and normal tissues were analyzed through the Wilcox test. The *p*-value was adjusted with the false discovery rate (FDR), and the filter criteria were FDR < 0.05 and |log_2_ fold-change [FC]| > 1. The expression of CD248 between groups was analyzed through the *t*-test and visualized with the GraphPad Prism 8 (GraphPad Software, San Diego, CA, USA). A body map of the expression of CD248 was obtained from the Gene Expression Profiling Interactive Analysis (GEPIA) website (http://gepia.cancer-pku.cn/), and the expression median was normalized to transcripts per million (TPM).

### Qualitative Evaluation of the Expression of CD248 in RCC

Paraffin-embedded tissue microarrays (Outdo Biotech, Shanghai, China) were deparaffinized, rehydrated, and treated with 3% hydrogen peroxide for 10 min to inhibit endogenous peroxidase activity. Heat-mediated retrieval of antigens was performed in citrate buffer for 2 min. After being blocked with 5% bovine serum albumin (BSA) for 30 min, slides were incubated with rabbit anti-human CD248 primary antibody (1:2,000, ab204914, Abcam, MA, USA) overnight at 4°C. The immunodetection was performed using the standard rapid EnVision technique (Dako, Glostrup, Denmark). Subsequently, slides were washed in distilled water and counterstained with hematoxylin. Digital images for qualitative evaluation were obtained using an optical microscope (BX51, Olympus, Tokyo, Japan).

### The Prognostic Value Analysis of CD248 in RCC

Patients with TCGA-RCC were divided into high expression and low expression groups according to the median expression level of CD248. Then, Kaplan–Meier survival analysis and receiver operating characteristic (ROC) analysis were performed to evaluate the prognostic value and the predictive accuracy of CD248, respectively. Univariate and multivariate Cox regression analyses were employed to identify the independent prognostic factors of RCC. *p* < 0.05 was considered statistically significant.

### The Construction and Validation of the CD248-based Prognostic Signature

Patients with TCGA-RCC with complete clinical information (*n* = 246) were used as a training cohort, and patients with TCGA-clear cell RCC (ccRCC) with certain clinical information (i.e., age, histological grade, pathological stage, and M status) were selected as a testing cohort (*n* = 489). The training cohort was used to construct a CD248-based prognostic signature, and the testing cohort was used to confirm its performance. The survival R package was adopted to construct the present signature by integrating clinicopathological variables [i.e., age, gender, histological grade, pathological stage, and tumor-node-metastasis (TNM) status] with the expression level of CD248. To avoid overfitting, clinicopathological variables that correlated highly with CD248 were deleted during data analysis. Then, Cox proportional hazards regression was used to build a prognostic risk model, and the regression coefficients were used as weight variables of the model. The risk score of each patient was calculated using the following formula, and the median was employed to separate both cohorts into different risk groups (13):

$$\begin{array}{l} Risk\;score\;=\overset{n}{\underset{i=1}{\sum }}coefficient\;{\left(gene\;i\right)}^{*}Expression\;value\;of\;\left(gene\;i\right) \end{array}$$

The survival and ROC analysis were performed as mentioned. To visualize the present signature, a nomogram was constructed by the rms R package.

### The Correlation Between the CD248-based Signature and TIICs

The tumor purity and the immune score of patients with TCGA-RCC were assessed through the ESTIMATE R package as previously reported (14). The relative fraction of 22 types of TIICs in each sample was quantified by the CIBERSORT method and the LM22 signature matrix (15, 16). The algorithm ran at 100 permutations with a threshold of *p* < 0.05 to select eligible patients (17). The correlation between the risk score and TIICs was analyzed with the Pearson correlation coefficient test, and the impact of TIICs on clinicopathological features was analyzed with the Wilcox test and the Kruskal–Wallis test. The box plot was prepared with the beeswarm R package.

### The Correlation Between the CD248-based Signature and the Tumor Mutation Burden

Masked somatic mutation data (VarScan) of RCC were retrieved from the TCGA portal. Non-synonymous somatic mutations of each patient were counted by the Perl software (https://www.perl.org/). Then, we used 38 Mb as the estimate of the exome size and calculated the TMB (i.e., mutation density) with the following formula: TMB = total mutation frequency/38. The Wilcox test was adopted to evaluate the relationship between the TMB and the risk score or clinicopathological features. *p* < 0.05 was considered statistically significant.

### The Correlation Between the CD248-based Signature and Immunomodulatory Molecules

Immune checkpoint molecules (i.e., PD1, CTLA4, LAG3, TIM3, BTLA, and VSIR) and immunostimulatory molecules (i.e., CD28, CD27, TNFRSF4, TNFRSF9, and TNFRSF18) play important roles in immunoregulation. In the present study, the expression level of the aforementioned molecules between two risk groups was analyzed with the Wilcox test. The Kaplan–Meier survival analysis was performed using the R software, and the median expression level was used as the cut-off value. *p* < 0.05 was considered statistically significant.

### The Weighted Gene Co-expression Network Analysis and the Enrichment Analysis

Differentially expressed genes (DEGs) co-expressed with CD248 were selected through the Pearson correlation coefficient test and visualized with the pheatmap R package. Filter criteria were |correlation coefficient| > 0.5 and *p* < 0.001. The WGCNA was employed to identify CD248 co-expressed modules. Briefly, the gradient method was used to screen out the appropriate power value with an independence degree of 0.9. The cluster analysis was performed to construct a dendrogram, and the module-trait heatmap was painted to identify the phenotype (clinic trait) and the highly correlated expression set (module). Finally, the interested modules were visualized with Cytoscape 3.6.0 and analyzed with GO and KEGG enrichment analysis. FDR < 0.05 was used as the threshold.

### CD248 Correlated miRNA Analysis

The software package edgeR was adopted to identify the differentially expressed miRNA (DEmiRNA) between tumor and normal tissues. The filter criteria were FDR < 0.05 and log_2_ FC > 1. Subsequently, CD248-correlated DEmiRNA and survival-related DEmiRNA were selected through the Pearson correlation coefficient test and the Kaplan–Meier survival analysis, respectively. Filter criteria were |correlation coefficient| > 0.5 and *p* < 0.001. The intersection of those two kinds of DEmiRNA was visualized with the Venn diagram and the heatmap.

## Results

### The Overexpression of CD248 in Tissues With RCC

**Figure 1:**
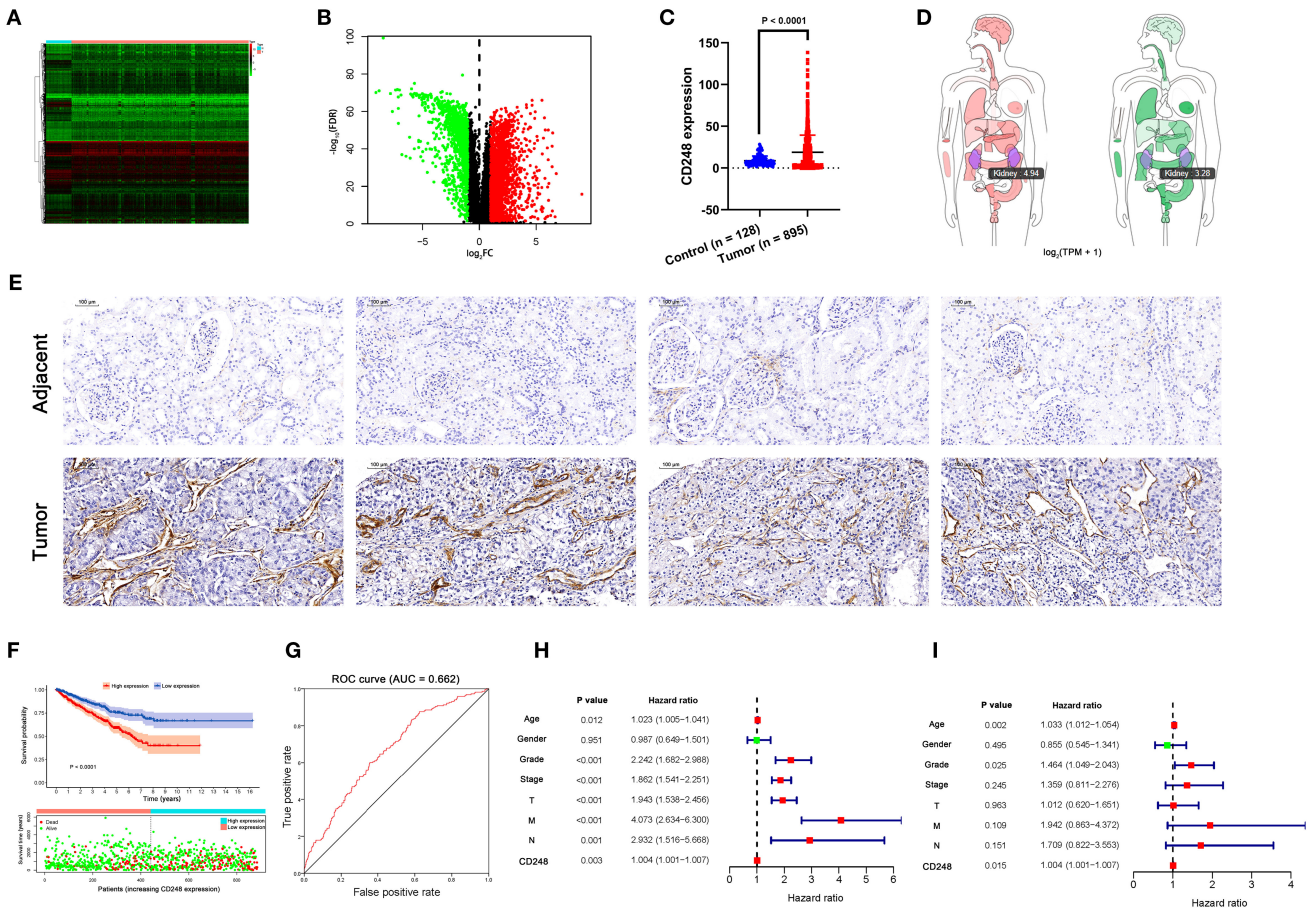
The expression and the prognostic value of CD248. **(A)** A heat map of DEGs. Green to red spectrum indicates low to high gene expression. **(B)** A volcano plot of DEGs. Red, green, and black dots represent upregulated, downregulated, and unchanged genes, respectively. **(C)** The overexpression of CD248 in tissues with RCC **(D)** A body map of the expression of CD248 **(E)** Immunohistochemical qualitative evaluation of CD248 in RCC. Scale bar = 100 μm. **(F)** The prognostic value of CD248. Ninety-five percent confidence interval is shown as light-colored background. **(G)** The ROC curve of CD248 **(H)** Univariate Cox regression analysis of CD248 **(I)** Multivariate Cox regression analysis of CD248. Red and green dots represent variables with hazard ratio > 1 and ≤ 1, respectively. *p* < 0.05 was considered statistically significant.

Based on the TCGA-RCC data set, 3,086 DEGs were obtained, among which 1,127 genes were downregulated, and 1,959 genes were upregulated in tissues with RCC compared with the normal (FDR < 0.05, |log_2_ FC| > 1, Figures 1A,B). Then, the overexpression of CD248 in tissues with RCC was identified (*p* < 0.0001, Figure 1C and Supporting Data 1). The body map of CD248 showed that the median expression level in RCC was 4.94, which was much higher than 3.28 in the normal kidney (Figure 1D). The results of the immunohistochemical staining indicated overexpression of CD248 in RCC instead of the adjacent normal tissues (Figure 1E). As shown in Figure 1F, the prognosis was poor in the high-expression CD248 group than in the low-expression group (*p* < 0.0001). Precisely, the overall survival (OS) rate at 5-year for the high-expression group was 58.8%, and the corresponding rate for the low-expression group was 75.3%. The area under the ROC curve (AUC) was 0.662, suggesting that CD248 could accurately predict the OS of patients with RCC (Figure 1G). Additionally, univariate and multivariate Cox regression analyses revealed that CD248 could serve as an independent prognostic factor for RCC (*p* < 0.05, Figures 1H,I).

### The Prognostic Value of the CD248-based Signature

The training cohort was adopted to construct a CD248-based signature. After deleting clinicopathological variables that would overfit the signature, coefficients were estimated through multivariate Cox regression. Subsequently, the risk score for each patient was calculated with the following formula:

Risk score = (0.0291 × age) + (0.4245 × histological grade) + (0.3303 × pathological stage) + (0.6492 × M status) + (0.0038 × the expression level of CD248)

**Figure 2:**
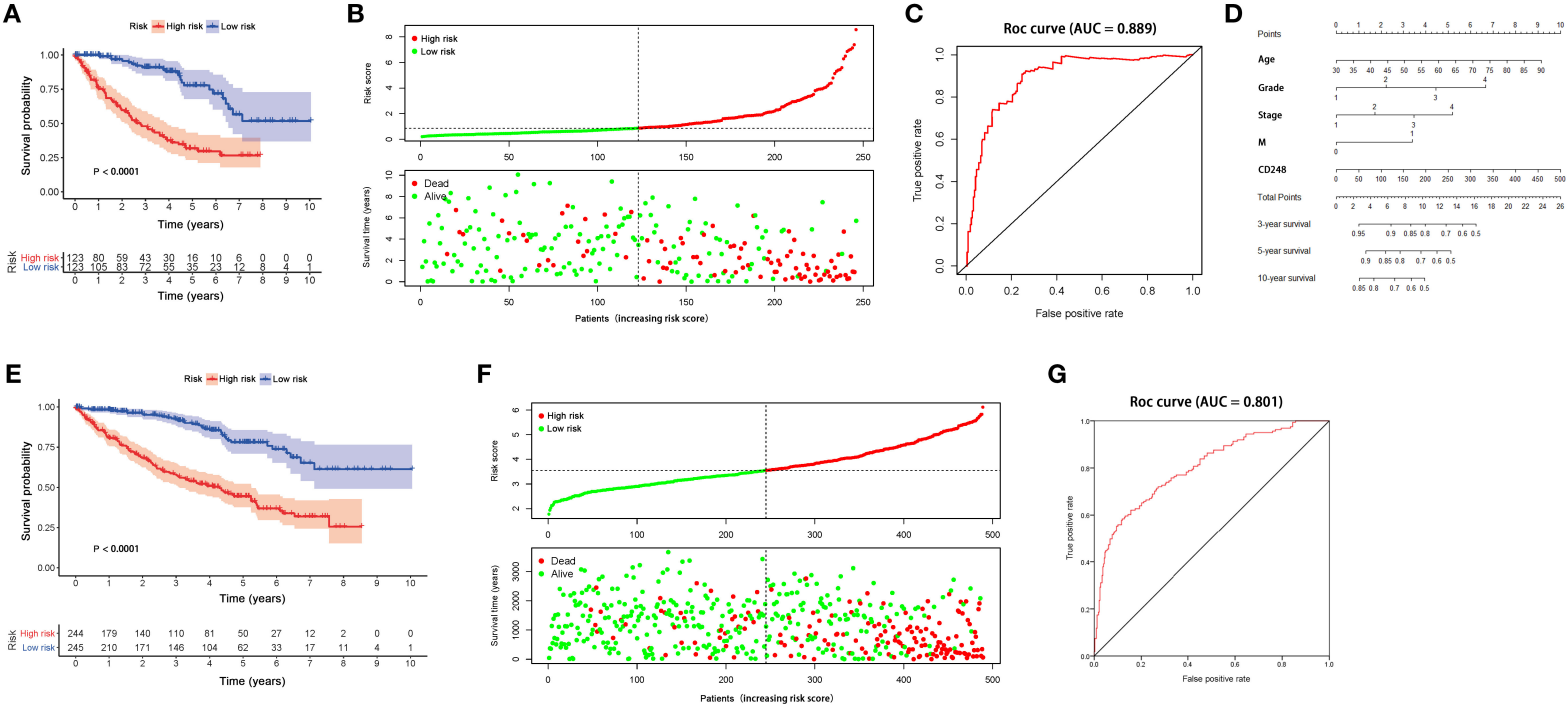
The prognostic value of CD248-based signature **(A)** The Kaplan–Meier curve of a training cohort **(B)** The distribution of the risk score and the survival status of each patient in the training cohort **(C)** The ROC curve of the present signature in the training cohort **(D)** A nomogram of the present signature **(E)** The Kaplan–Meier curve of the testing cohort **(F)** The distribution of the risk score and the survival status of each patient in the testing cohort **(G)** The ROC curve of the present signature in the testing cohort. Ninety-five percent confidence interval is shown as light-colored background. *p* < 0.05 was considered statistically significant.

According to the median risk score 0.8618, individuals in the training cohort were sorted into a high-risk (*n* = 123) and a low-risk group (*n* = 123). The Kaplan–Meier survival analysis showed that the prognosis was worse in the high-risk group than in the low-risk group (*p* < 0.0001, Figure 2A). The OS rate at 5 years for the high-risk and the low-risk groups was 31.7 and 77.7%, respectively. Then, we ranked patients with the risk score and analyzed their survival status. As shown in Figure 2B, a large amount of death was distributed in the high-risk group. The AUC value for the present signature was 0.889 (Figure 2C). To facilitate clinical utility, a nomogram to predict the prognosis of RCC at 3, 5, and 10 years was prepared accordingly (Figure 2D).

A testing cohort was used to verify the accuracy of the present signature. As shown in Figures 2E,F, the survival status of patients with ccRCC differed significantly between the two risk groups (*p* < 0.05). The survival rates at 3 and 5 years in the high-risk group were 58.1 and 44.4%, respectively, while the corresponding rates in the low-risk group were 92.1 and 78.0%, respectively. Moreover, the AUC value of the present signature was 0.801 in the testing cohort (Figure 2G).

### The Correlation Between the Present Signature and the Tumor Immune Microenvironment

**Figure 3:**
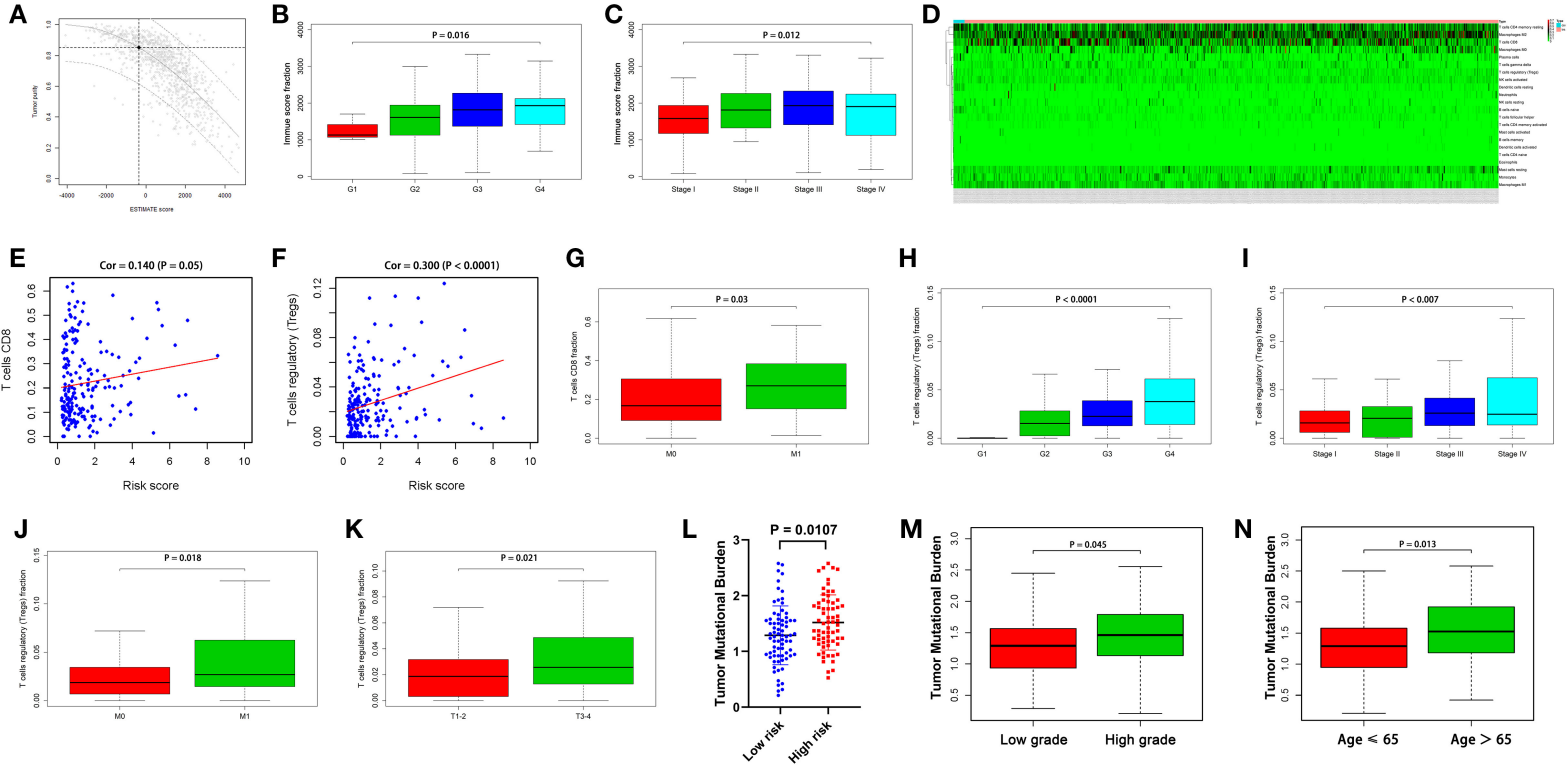
The relationship between the present signature and the tumor immune microenvironment **(A)** The tumor purity assessed with the ESTIMATE algorithm **(B)** The immune score and deteriorated histological grade **(C)** The immune score and advanced pathological stage **(D)** TIICs assessed with the CIBERSORT algorithm **(E)** The correlation between the risk score and CD8^+^ T cells **(F)** The correlation between the risk score and Tregs **(G)** CD8^+^ T cells fraction and the metastasis of RCC **(H)** Tregs fraction and deteriorated histological grade **(I)** Tregs fraction and advanced pathological stage **(J)** Tregs fraction and the metastasis of RCC **(K)** Tregs fraction and the tumor size **(L)** Increase in the TMB in the high-risk group **(M)** The TMB and deteriorated histological grade **(N)** Increase in the TMB with age. *p* < 0.05 was considered statistically significant.

The ESTIMATE and CIBERSORT algorithms were employed to assess tumor purity and infiltrating immune cells (Figures 3A,D). As shown in Figures 3B,C, an increased immune score was related to deteriorated histological grade and pathological stage (*p* < 0.05). The fraction of CD8^+^ T cells and regulatory T cells (Tregs) was positively related to the risk score generated by the present signature (*p* < 0.05, Figures 3E,F). Furthermore, a high proportion of CD8^+^ T cells and Tregs could lead to a poor prognosis of patients with RCC, accompanied by increased histological grade, bad pathological stage, and tumor metastasis (*p* < 0.05, Figures 3G–K).

The TMB is a vital factor affecting tumor immune response and immunotherapy. In the present study, with the increase of the risk score generated by the present signature, the TMB significantly increased (*p* < 0.05, Figure 3L). Besides, higher TMB was associated with worse histological grade, and the value of the TMB increased with the age of the patients (*p* < 0.05, Figures 3M,N).

### The Correlation Between the Present Signature and Immunomodulatory Molecules

**Figure 4:**
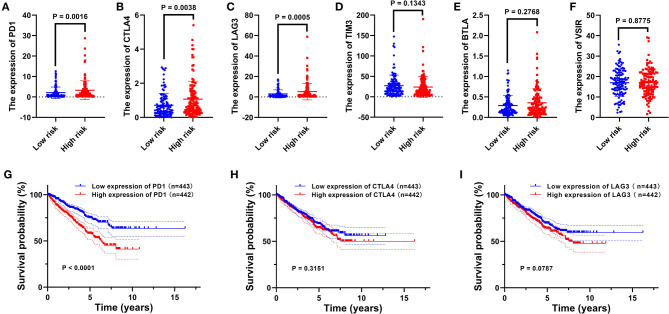
The relationship between the present signature and immune checkpoints. Expression analysis of PD1 **(A)**, CTLA4 **(B)**, LAG3 **(C)**, TIM3 **(D)**, BTLA **(E)**, and VSIR **(F)** between two risk groups. The Kaplan–Meier curve of PD1 **(G)**, CTLA4 **(H)**, and LAG3 **(I)**. The median expression level was used as the cut-off value. *p* < 0.05 was considered statistically significant.

**Figure 5:**
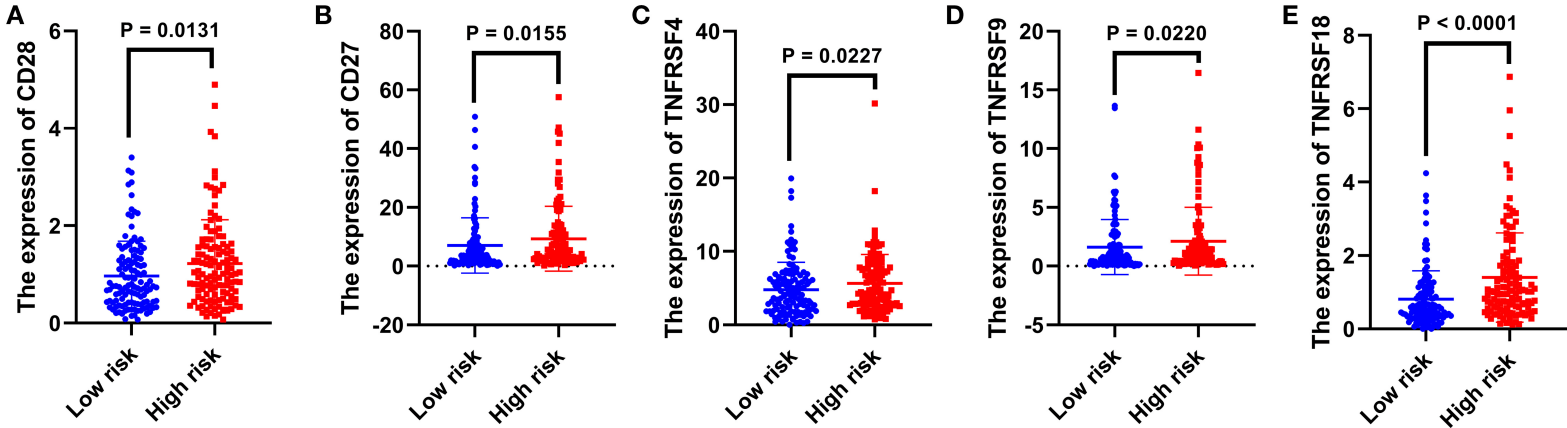
The relationship between the present signature and immunostimulatory molecules. The expression analysis of CD28 **(A)**, CD27 **(B)**, TNFRSF4 **(C)**, TNFRSF9 **(D)**, and TNFRSF18 **(E)** between two risk groups. *p* < 0.05 was considered statistically significant.

The expression level of immunomodulatory molecules was regarded as a promising indicator to guide immunotherapy. We found that the expression of some immune checkpoint molecules (i.e., PD1, CTLA4, and LAG3) increased in the high-risk group (*p* < 0.01, Figures 4A–C), while others (i.e., TIM3, BTLA, and VSIR) remained unchanged between the two risk groups (*p* > 0.05, Figures 4D–F). The survival analysis indicated that highly expressed PD1 combined with a bad prognosis (*p* < 0.0001, Figure 4G), while the expression level of CTLA4 and LAG3 did not significantly affect the OS of patients with RCC (*p* > 0.05, Figures 4H,I). In addition to immune checkpoints, the expression of immunostimulatory molecules has been investigated as well. As shown in Figure 5, the expression of commonly detected immunostimulatory molecules, such as CD28, CD27, TNFRSF4, TNFRSF9, and TNFRSF18, was upregulated in the high-risk group (*p* < 0.05).

### The Weighted Gene Co-expression Network Analysis and the Enrichment Analysis of CD248 Co-expressed Genes

**Figure 6:**
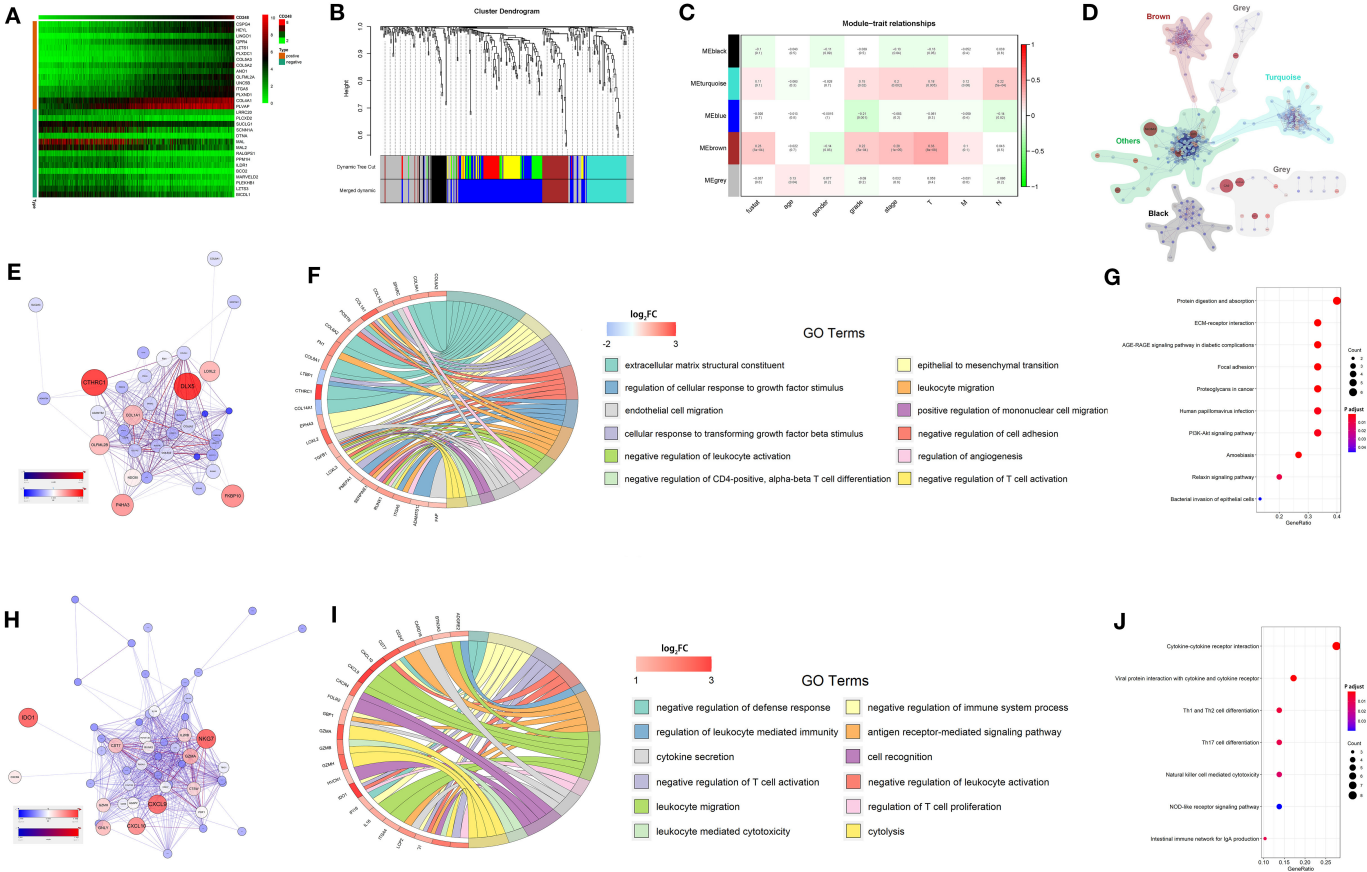
The WGCNA and enrichment analysis of CD248 co-expressed genes **(A)** The heatmap of CD248 co-expressed genes **(B)** The identification of co-expressed modules **(C)** The module-trait heatmap **(D)** The interaction among co-expressed modules. The co-expressed network **(E)**, GO enrichment analysis **(F)**, and KEGG enrichment analysis **(G)** of brown modules. The co-expressed network **(H)**, GO enrichment analysis **(I)**, and KEGG enrichment analysis **(J)** of turquoise modules. FDR < 0.05 was considered as statistically significant.

Through the Pearson correlation coefficient test, 334 DEGs co-expressed with CD248 were selected (|correlation coefficient| > 0.5 and *p* < 0.001, Supporting Data 2). The top 15 DEGs that positively and negatively correlated with CD248 were adopted to develop a co-expressed heatmap (Figure 6A). Subsequently, we identified five distinct CD248 co-expressed modules through the WGCNA (Figure 6B). The module-trait heatmap indicated that brown and turquoise modules were significantly associated with the progression of RCC (*p* < 0.05, Figure 6C). Then, intramodular and extramodular interactions were visualized, especially the modules marked with brown and turquoise (Figures 6D,E,H). With the co-expressed network, several hub genes with maximum intramodular connectivity were identified, which might play a vital role in the progression of RCC.

To explore the underlying mechanism of the brown and turquoise modules on the progression of RCC, GO and KEGG enrichment analysis was performed. As shown in Figures 6F,I, “negative regulation of defense response,” “negative regulation of immune system process,” “negative regulation of leukocyte activation,” “negative regulation of cell adhesion,” etc. immunosuppressive GO terms were significantly enriched (FDR < 0.05). Then, KEGG enrichment analysis showed that several immunomodulatory signaling pathways were significantly enriched, including “PI3K-Akt signaling pathway,” “cytokine–cytokine receptor interaction,” “Th1 and Th2 cell differentiation,” “natural killer cell mediated cytotoxicity,” and “NOD-like receptor signaling pathway” (FDR < 0.05, Figures 6G,J).

### The Correlation Between CD248 and miRNA

**Figure 7:**
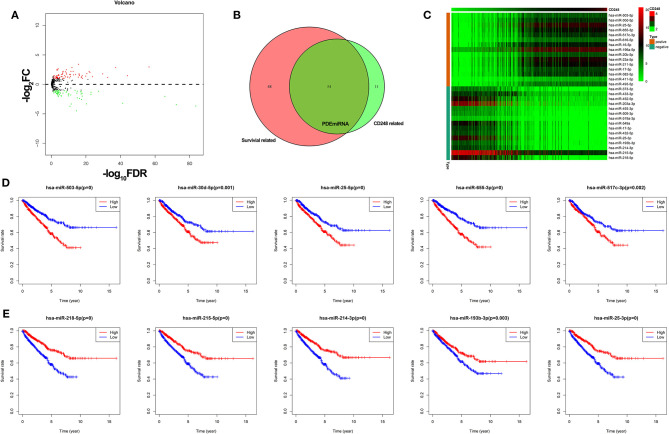
The correlation between CD248 and miRNA **(A)** The volcano plot of DEmiRNA. Red, green, and black dots represent upregulated, downregulated, and unchanged genes, respectively. **(B)** The Venn diagram of PDEmiRNA **(C)** The heatmap of the top 15 CD248-correlated PDEmiRNA **(D)** The Kaplan–Meier curve of the top five PDEmiRNA that positively correlated with CD248 **(E)** The Kaplan–Meier curve of the top five PDEmiRNA that negatively correlated with CD248. The median expression level of PDEmiRNA was used as the cut-off value. *p* < 0.05 was considered statistically significant.

Through the edgeR package, 138 DEmiRNA were obtained (FDR < 0.05, |log_2_ FC| > 1, Figure 7A), among which 65 DEmiRNA were significantly correlated with the expression of CD248 (correlation coefficient > 0.5 and *p* < 0.001), and 102 DEmiRNA were related with the survival of RCC (*p* < 0.05). Then, 54 CD248-correlated DEmiRNA that related with the prognosis of RCC (PDEmiRNA) were identified (Figure 7B and Supporting Data 3), and a heatmap of the top 15 PDEmiRNA that positively and negatively correlated with CD248 was developed (Figure 7C). Additionally, the top 5 PDEmiRNA that positively correlated with the expression of CD248 (i.e., hsa-miR-503-5p, hsa-miR-30d-5p, hsa-miR-25-5p, hsa-miR-655-3p, and hsa-miR-517c-3p) reflected a bad survival outcome (*p* < 0.05, Figure 7D). However, the top five negatively correlated PDEmiRNA (i.e., hsa-miR-218-5p, hsa-miR-215-5p, hsa-miR-214-3p, hsa-miR-193b-3p, and hsa-miR-25-3p) indicated a better prognosis (*p* < 0.05, Figure 7E).

## Discussion

The incidence of RCC has increased steadily by about 1% per year, and the high mortality rate remains unchanged worldwide (18). Despite substantial improvements in available treatment options, the 5-year survival rate for advanced/metastatic RCC is <23% (19). In fact, about 30–50% of patients with RCC have missed the best surgical opportunity due to the incidence of the occult (20). Immunotherapy is an emerging method to prolong the OS of RCC. However, the low response rate results in an unsatisfied clinical outcome. Accordingly, various biomarkers have been suggested to assist in the early diagnosis and to guide treatment selection. Chen et al. reported that miR-30a-3p could inhibit the invasion of RCC and serve as a new prognostic marker (21). miR-142-3p has also proved to be involved with tumorigenesis and the development of RCC (22). In addition to miRNAs, mRNAs including HHLA2 and syntaxin 6 were associated with decreased survival, and corresponding inhibitors held promise as a novel therapy against RCC (23, 24). However, the functional effect of a single gene in the progression of RCC is relatively weak. Identifying sensitive and specific indicators to improve diagnostic and therapeutic efficiency is still urgently needed.

Recently, a great amount of evidence indicates that TEMs have a broad influence in complicated cross-talk between tumor cells and the tumor microenvironment, which would lead to the progression of tumors (25, 26). Thus, TEMs appear to be promising candidates for the early detection of tumors, monitoring, and treatment. As an important part of TEMs, the biological function of CD248 in RCC remains unclear. In the present study, the expression level of CD248 in tissues with RCC was evaluated through the TCGA data set and confirmed in external clinical specimens. Through the qualitative evaluation of immunohistochemistry, we found the overexpression of CD248 in RCC compared with adjacent normal kidney tissues. In addition, highly expressed CD248 was associated with bad prognosis. CD248 could also serve as an independent prognostic factor to predict the OS in patients with RCC, and the predictive accuracy (AUC = 0.662) was regarded as acceptable (27). These findings indicate that the expression level of CD248 could be a new early diagnostic and prognostic marker for RCC. Subsequently, to establish a clinically stratifying system to improve the diagnostic efficiency, we constructed a CD248-based prognostic signature. This signature could stratify patients with TCGA-RCC into two risk groups with statistically different survival outcomes, and the predictive accuracy (AUC = 0.889) was deemed to be excellent (27). The reliability of the present signature was further verified in a testing cohort, and a nomogram was prepared to facilitate its clinical application.

The tumor immune microenvironment, comprising infiltrating immune cells and immune-related proteins (IRPs), has emerged as an important player in the progression of tumors (28, 29). In the present study, we explored the correlation between the present signature and the dysfunctional immune microenvironment. We found that infiltration of Tregs in RCC significantly increased with the risk score generated by the present signature, and a high immune score and high infiltration of Tregs accompanied by bad histological grade, advanced pathological stage, and more chance of metastasis in previous studies (30). Additionally, CD8^+^ cytotoxic T lymphocytes (CTLs) were positively correlated with the risk score; however, increased CTLs resulted in the metastasis of RCC instead of the killing effect. Thus, we speculate the CTLs-mediated anti-tumor response is counterbalanced by strong immunosuppression of Tregs, which consequentially facilitate the survival and metastasis of cancer cells (31, 32).

The killing efficacy of CTLs is also directly or indirectly regulated by IRPs and TMB (33–35). The immune checkpoints (i.e., PD1, CTLA4, and LAG3) were upregulated in the high-risk group, which might induce the depletion of CTLs, the tumor immune escape, and poor survival outcome. In addition, commonly detected immunostimulatory molecules (i.e., CD28, CD27, TNFRSF4, TNFRSF9, and TNFRSF18) were upregulated in the high-risk group. The TMB—a surrogate for neoantigen level and malignant degree—increased with the risk score generated by the present signature. Based on the immune landscape of high-risk patients, once the immune suppression of CTLs is removed, the self–anti-tumor immune response would be expanded, and high-risk patients might benefit from immunotherapy. Therefore, the present signature could not only contribute to the early diagnosis of patients with RCC but also assist clinicians to screen immunotherapeutic-sensitive patients. Inevitably, a large-scale prospective validation of clinical benefits before widespread adoption is necessary (36).

To explore possible functions of CD248 in RCC, the WGCNA and enrichment analysis were performed. The results suggested that CD248 co-expressed genes could be divided into five modules, among which the brown and turquoise modules were significantly associated with the progression of RCC. Then, the identified prognostic-related modules were analyzed with the GO and the KEGG algorithm. As a result, several immunosuppressive GO terms were significantly enriched, including the negative regulation of leukocyte activation, migration, adhesion, and differentiation, which might provide insight into the depletion of CTLs mentioned above. More accurately, tumor stroma might play an important role in negative immunoregulation since the hub genes (i.e., CTHRC1, COL1A1, LOXL2, P4HA3, and FKBP10) related to immunosuppressive GO terms usually participate in collagen formation. With the immunosuppressive landscape, the expression of CTL effectors (i.e., GZMA, GZMH, and GNLY) would be inhibited. Meanwhile, chemokines (i.e., CXCL9 and CXCL10), inflammatory factors [i.e., interleukin-16 (IL-16), IL-2, and IFI16], and relevant signaling pathways might negatively regulate the activation and migration of CTLs. After verifying them in studies *in vitro* or *in vivo*, novel diagnostic and therapeutic targets might be proposed.

Furthermore, the correlation between CD248 and miRNA was explored, which would be valuable to reveal the potential mechanism of the transcriptomic regulation. Interestingly, the PDEmiRNA that positively or negatively correlated with the expression of CD248 could reflect a bad or good survival outcome, respectively. Therefore, the downregulation of protective PDEmiRNA (CD248 negatively correlated) might contribute to the risk of the overexpression of the gene (i.e., CD248), leading to a poor prognosis. This result further emphasized the importance of CD248 in RCC.

In summary, we identified a valuable biomarker and constructed a reliable prognostic signature that can precisely predict the prognosis of patients with RCC. Additionally, the present signature can effectively screen outpatients with RCC suitable for immunotherapy. The WGCNA, enrichment analysis, and miRNA correlation analysis revealed possible functions and the regulation of the expression of CD248, which may contribute to explain CD248-mediated progression of RCC and provide potential diagnostic and therapeutic targets.

## Data Availability Statement

Publicly available datasets were analyzed in this study. This data can be found at: https://portal.gdc.cancer.gov.

## Author Contributions

KZ, CX, and SL: data acquisition, data analysis, and writing the original draft. KZ, CX, SL, YJ, XZ, SM, and YL: methodology, data interpretation, writing the review, and editing. FY, YW, PM, and CS: literature research and immunohistochemical assay. DH, WW, and WQ: conceptualization, design, and project administration. All authors approved the submitted version.

## Conflict of Interest

The authors declare that the research was conducted in the absence of any commercial or financial relationships that could be construed as a potential conflict of interest.
