# Supplementary material for: The Diagnostic and Immunotherapeutic Value of CD248 in Renal Cell Carcinoma
Source: Front Oncol. 2021 Mar 12;11:644612. doi: 10.3389/fonc.2021.644612 (PMC8006336; doi:10.3389/fonc.2021.644612)
Supplement: Supplementary file 1 [file Table_1.DOCX]

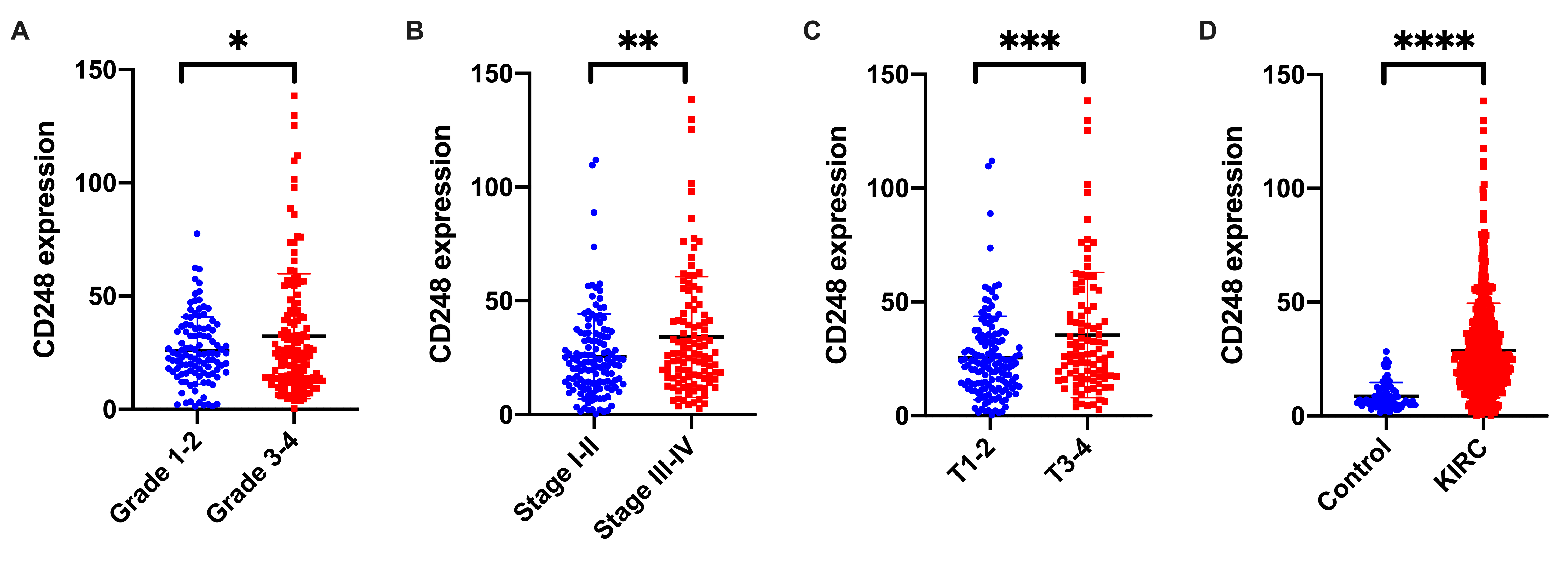


**Supporting data 1** CD248 expression based on different RCC characteristics. **(A)** CD248 overexpressed in RCC group with high histological grade. **(B)** CD248 overexpressed in RCC group with deteriorated pathological stage. **(C)** CD248 overexpressed in RCC group with large tumor size. **(D)** CD248 overexpressed in kidney renal clear cell carcinoma (KIRC). ^*^*P* < 0.05, ^**^*P* < 0.01, ^***^*P* < 0.001, ^****^*P* < 0.0001.
