# Supplementary material for: The Diagnostic and Immunotherapeutic Value of CD248 in Renal Cell Carcinoma
Source: Front Oncol. 2021 Mar 12;11:644612. doi: 10.3389/fonc.2021.644612 (PMC8006336; doi:10.3389/fonc.2021.644612)
Supplement: Supplementary file 2 [file Table_2.DOCX]

**Supporting data 2** CD248 co-expressed DEGs

| **Gene 1** | **Gene 2** | **Pearson correlation coefficient** | ***P* value** |
| --- | --- | --- | --- |
| CD248 | AMACR | -0.52 | 6.26E-72 |
| CD248 | CYP4X1 | 0.548 | 2.43E-81 |
| CD248 | BCO2 | -0.602 | 4.44E-102 |
| CD248 | HVCN1 | 0.582 | 1.32E-93 |
| CD248 | ELMO1 | 0.761 | 3.66E-194 |
| CD248 | GRIK3 | 0.7 | 2.56E-151 |
| CD248 | LY6H | 0.506 | 1.01E-67 |
| CD248 | FKBP10 | 0.599 | 8.74E-101 |
| CD248 | CPNE5 | 0.714 | 5.09E-160 |
| CD248 | CYGB | 0.828 | 3.43E-258 |
| CD248 | ACAN | 0.739 | 2.43E-177 |
| CD248 | SCNN1A | -0.573 | 2.27E-90 |
| CD248 | SLC2A5 | 0.522 | 1.59E-72 |
| CD248 | P4HA1 | 0.575 | 4.51E-91 |
| CD248 | TMC4 | -0.521 | 3.35E-72 |
| CD248 | ACOT11 | -0.512 | 1.38E-69 |
| CD248 | EGLN3 | 0.655 | 1.28E-126 |
| CD248 | MPC1 | -0.511 | 5.03E-69 |
| CD248 | CTHRC1 | 0.606 | 1.09E-103 |
| CD248 | GJA1 | 0.659 | 1.84E-128 |
| CD248 | GBP1 | 0.617 | 1.85E-108 |
| CD248 | NRARP | 0.826 | 6.03E-256 |
| CD248 | GMFG | 0.7 | 1.05E-151 |
| CD248 | FBXO2 | -0.502 | 2.01E-66 |
| CD248 | CAV1 | 0.683 | 2.31E-141 |
| CD248 | LDHA | 0.533 | 3.49E-76 |
| CD248 | LRRC17 | 0.779 | 5.87E-209 |
| CD248 | NR2F1 | 0.601 | 2.69E-101 |
| CD248 | TRPV2 | 0.691 | 6.37E-146 |
| CD248 | ANO1 | 0.85 | 1.07E-286 |
| CD248 | SEMA6B | 0.722 | 9.59E-166 |
| CD248 | COL5A2 | 0.857 | 1.28E-296 |
| CD248 | HEYL | 0.884 | 0 |
| CD248 | BTN3A3 | 0.505 | 3.39E-67 |
| CD248 | GABRD | 0.815 | 2.92E-244 |
| CD248 | HAPLN3 | 0.652 | 8.03E-125 |
| CD248 | PPP1R3C | 0.594 | 9.95E-99 |
| CD248 | LOX | 0.694 | 4.33E-148 |
| CD248 | C1QL1 | 0.514 | 3.72E-70 |
| CD248 | PRDM1 | 0.749 | 1.30E-184 |
| CD248 | NETO2 | 0.682 | 5.18E-141 |
| CD248 | ERO1A | 0.528 | 1.80E-74 |
| CD248 | PRR16 | 0.796 | 3.49E-225 |
| CD248 | NES | 0.783 | 1.39E-212 |
| CD248 | KSR1 | 0.598 | 3.93E-100 |
| CD248 | EDN1 | 0.599 | 9.86E-101 |
| CD248 | FN1 | 0.664 | 3.11E-131 |
| CD248 | IL18BP | 0.587 | 1.07E-95 |
| CD248 | APOLD1 | 0.742 | 1.12E-179 |
| CD248 | DTNA | -0.575 | 5.95E-91 |
| CD248 | LAMA4 | 0.689 | 7.17E-145 |
| CD248 | GABRE | 0.542 | 3.16E-79 |
| CD248 | FOXJ3 | 0.542 | 3.60E-79 |
| CD248 | COL1A2 | 0.757 | 7.36E-191 |
| CD248 | COL4A1 | 0.842 | 1.64E-276 |
| CD248 | TNFRSF1B | 0.542 | 4.52E-79 |
| CD248 | TGFB1 | 0.703 | 1.93E-153 |
| CD248 | NKG7 | 0.557 | 2.90E-84 |
| CD248 | AFAP1L1 | 0.696 | 5.81E-149 |
| CD248 | LTBP1 | 0.627 | 5.19E-113 |
| CD248 | PRF1 | 0.613 | 1.38E-106 |
| CD248 | MAP4K4 | 0.52 | 4.93E-72 |
| CD248 | P2RY8 | 0.728 | 2.01E-169 |
| CD248 | ACSM1 | -0.544 | 5.25E-80 |
| CD248 | PLEKHO1 | 0.589 | 1.07E-96 |
| CD248 | ST8SIA4 | 0.681 | 1.49E-140 |
| CD248 | OCLN | -0.563 | 1.83E-86 |
| CD248 | CHSY3 | 0.782 | 1.93E-211 |
| CD248 | HILPDA | 0.679 | 2.06E-139 |
| CD248 | TBX21 | 0.537 | 1.21E-77 |
| CD248 | PRND | 0.557 | 2.43E-84 |
| CD248 | DEPP1 | 0.756 | 3.24E-190 |
| CD248 | PLEKHB1 | -0.63 | 2.34E-114 |
| CD248 | TOX2 | 0.679 | 4.37E-139 |
| CD248 | PLIN2 | 0.553 | 5.89E-83 |
| CD248 | SLC35G2 | 0.595 | 7.75E-99 |
| CD248 | ADGRE2 | 0.511 | 3.32E-69 |
| CD248 | NRP2 | 0.668 | 2.42E-133 |
| CD248 | CACNA1H | 0.519 | 1.70E-71 |
| CD248 | ADSSL1 | 0.6 | 3.04E-101 |
| CD248 | ADM | 0.694 | 1.08E-147 |
| CD248 | TUBB2B | -0.515 | 1.90E-70 |
| CD248 | BTNL9 | 0.625 | 4.70E-112 |
| CD248 | COL8A1 | 0.664 | 5.76E-131 |
| CD248 | PLVAP | 0.842 | 1.16E-275 |
| CD248 | CES4A | 0.524 | 2.76E-73 |
| CD248 | DYSF | 0.752 | 7.16E-187 |
| CD248 | PNCK | 0.59 | 7.69E-97 |
| CD248 | ARHGAP25 | 0.646 | 6.98E-122 |
| CD248 | SLFN11 | 0.672 | 1.77E-135 |
| CD248 | SLC6A3 | 0.653 | 2.14E-125 |
| CD248 | 3-Mar | 0.582 | 7.61E-94 |
| CD248 | FAM198B | 0.668 | 6.02E-133 |
| CD248 | TNFSF13B | 0.505 | 2.47E-67 |
| CD248 | ATP6V1FNB | -0.529 | 5.96E-75 |
| CD248 | PODXL | 0.661 | 2.23E-129 |
| CD248 | STC2 | 0.769 | 6.63E-201 |
| CD248 | P4HA3 | 0.587 | 1.16E-95 |
| CD248 | PCSK6 | 0.643 | 1.74E-120 |
| CD248 | PPM1H | -0.585 | 3.99E-95 |
| CD248 | CDON | 0.538 | 1.05E-77 |
| CD248 | FAM49A | 0.649 | 2.33E-123 |
| CD248 | BATF3 | 0.577 | 9.40E-92 |
| CD248 | NDC80 | 0.518 | 3.17E-71 |
| CD248 | PROCR | 0.55 | 8.82E-82 |
| CD248 | ADAMTS12 | 0.621 | 2.39E-110 |
| CD248 | TLR3 | 0.527 | 4.84E-74 |
| CD248 | CES3 | 0.541 | 6.53E-79 |
| CD248 | ST6GALNAC3 | 0.537 | 2.24E-77 |
| CD248 | OR51E2 | 0.701 | 8.13E-152 |
| CD248 | OLFML2B | 0.838 | 1.93E-271 |
| CD248 | STARD9 | 0.516 | 1.14E-70 |
| CD248 | LZTS3 | -0.66 | 3.93E-129 |
| CD248 | ANGPT2 | 0.783 | 7.69E-213 |
| CD248 | TGFBI | 0.516 | 7.82E-71 |
| CD248 | KCNE3 | 0.703 | 2.47E-153 |
| CD248 | PRSS8 | -0.507 | 4.86E-68 |
| CD248 | CD247 | 0.549 | 1.48E-81 |
| CD248 | NGFR | 0.654 | 5.84E-126 |
| CD248 | HPCAL1 | 0.615 | 1.26E-107 |
| CD248 | FJX1 | 0.523 | 7.87E-73 |
| CD248 | FILIP1 | 0.703 | 3.72E-153 |
| CD248 | GZMB | 0.627 | 1.02E-112 |
| CD248 | SCN4B | 0.708 | 1.43E-156 |
| CD248 | EHD2 | 0.582 | 8.40E-94 |
| CD248 | SERPINB9 | 0.555 | 1.52E-83 |
| CD248 | GZMH | 0.553 | 4.45E-83 |
| CD248 | DGKD | 0.572 | 7.47E-90 |
| CD248 | LOXL2 | 0.715 | 6.44E-161 |
| CD248 | CA9 | 0.682 | 8.26E-141 |
| CD248 | KRBA1 | 0.58 | 3.55E-93 |
| CD248 | HAPLN1 | 0.619 | 2.21E-109 |
| CD248 | FOLR2 | 0.504 | 7.21E-67 |
| CD248 | CCDC181 | -0.507 | 6.86E-68 |
| CD248 | BARX2 | 0.575 | 3.24E-91 |
| CD248 | KIF26A | 0.515 | 1.74E-70 |
| CD248 | BICDL1 | -0.676 | 2.74E-137 |
| CD248 | COL5A1 | 0.771 | 6.23E-202 |
| CD248 | PDK1 | 0.532 | 7.18E-76 |
| CD248 | PIEZO2 | 0.699 | 4.51E-151 |
| CD248 | C5orf46 | 0.572 | 8.04E-90 |
| CD248 | KCNE4 | 0.823 | 3.57E-253 |
| CD248 | IL16 | 0.538 | 6.55E-78 |
| CD248 | CLEC2B | 0.638 | 8.84E-118 |
| CD248 | PLEKHG2 | 0.743 | 4.58E-180 |
| CD248 | ADAMTS7 | 0.545 | 4.75E-80 |
| CD248 | PPFIA4 | 0.53 | 3.62E-75 |
| CD248 | TMEM233 | 0.589 | 1.72E-96 |
| CD248 | CP | 0.631 | 1.02E-114 |
| CD248 | ITGA4 | 0.634 | 6.75E-116 |
| CD248 | KDF1 | -0.536 | 2.73E-77 |
| CD248 | VEGFA | 0.789 | 1.35E-218 |
| CD248 | ANGPTL4 | 0.723 | 7.00E-166 |
| CD248 | AP1M2 | -0.518 | 2.62E-71 |
| CD248 | CD36 | 0.723 | 2.63E-166 |
| CD248 | CXorf36 | 0.834 | 5.45E-266 |
| CD248 | HYKK | -0.529 | 6.90E-75 |
| CD248 | CTSW | 0.518 | 2.16E-71 |
| CD248 | MAL2 | -0.578 | 4.74E-92 |
| CD248 | PTP4A3 | 0.668 | 2.90E-133 |
| CD248 | IGSF21 | 0.605 | 5.12E-103 |
| CD248 | FAM43A | 0.519 | 1.42E-71 |
| CD248 | LZTS1 | 0.869 | 4.61799410197693e-314 |
| CD248 | RUNX1 | 0.56 | 1.89E-85 |
| CD248 | IFI16 | 0.607 | 4.56E-104 |
| CD248 | VWF | 0.726 | 4.22E-168 |
| CD248 | CD163L1 | 0.52 | 6.36E-72 |
| CD248 | FLT1 | 0.786 | 2.90E-215 |
| CD248 | HLX | 0.818 | 1.50E-247 |
| CD248 | TMCC1 | 0.588 | 3.90E-96 |
| CD248 | GZMA | 0.519 | 9.31E-72 |
| CD248 | LOXL3 | 0.537 | 1.56E-77 |
| CD248 | ANKRD2 | -0.548 | 2.95E-81 |
| CD248 | OLFML2A | 0.849 | 5.95E-285 |
| CD248 | ALDOC | 0.634 | 4.74E-116 |
| CD248 | EBF2 | 0.556 | 5.20E-84 |
| CD248 | CCND1 | 0.624 | 1.70E-111 |
| CD248 | TEK | 0.565 | 2.02E-87 |
| CD248 | HIGD1B | 0.726 | 2.29E-168 |
| CD248 | TSPAN2 | 0.547 | 9.34E-81 |
| CD248 | DLX5 | 0.792 | 3.76E-221 |
| CD248 | GPIHBP1 | 0.53 | 2.60E-75 |
| CD248 | OR51E1 | 0.791 | 6.86E-220 |
| CD248 | GDF6 | 0.633 | 8.46E-116 |
| CD248 | PCDH17 | 0.792 | 1.36E-220 |
| CD248 | CDCA2 | 0.64 | 5.34E-119 |
| CD248 | PLXND1 | 0.843 | 4.48E-277 |
| CD248 | C1orf162 | 0.526 | 6.17E-74 |
| CD248 | DNAH11 | 0.529 | 1.06E-74 |
| CD248 | S1PR4 | 0.514 | 3.59E-70 |
| CD248 | RASD2 | 0.742 | 1.32E-179 |
| CD248 | COL4A2 | 0.722 | 2.66E-165 |
| CD248 | P2RY1 | 0.714 | 2.68E-160 |
| CD248 | CDKN2B | 0.657 | 1.26E-127 |
| CD248 | ADAM19 | 0.757 | 6.80E-191 |
| CD248 | COL5A3 | 0.861 | 1.56E-301 |
| CD248 | ACKR3 | 0.767 | 1.33E-198 |
| CD248 | XAF1 | 0.519 | 1.34E-71 |
| CD248 | PMEPA1 | 0.738 | 6.68E-177 |
| CD248 | SH3GL2 | -0.507 | 6.41E-68 |
| CD248 | SAP30 | 0.625 | 7.99E-112 |
| CD248 | COL15A1 | 0.809 | 2.23E-237 |
| CD248 | PDIA5 | 0.646 | 5.38E-122 |
| CD248 | ITGA5 | 0.844 | 4.28E-279 |
| CD248 | TCAF2 | 0.653 | 3.38E-125 |
| CD248 | CSPG4 | 0.898 | 0 |
| CD248 | PRRG2 | -0.555 | 8.78E-84 |
| CD248 | TNFRSF4 | 0.772 | 8.78E-203 |
| CD248 | PAG1 | 0.567 | 5.29E-88 |
| CD248 | KCNAB1 | 0.677 | 5.42E-138 |
| CD248 | FAP | 0.617 | 2.02E-108 |
| CD248 | COL1A1 | 0.747 | 5.48E-183 |
| CD248 | SUCLG1 | -0.571 | 1.18E-89 |
| CD248 | MTCP1 | 0.516 | 8.45E-71 |
| CD248 | MCAM | 0.759 | 4.34E-192 |
| CD248 | JAG2 | 0.736 | 2.33E-175 |
| CD248 | STAMBPL1 | 0.525 | 2.12E-73 |
| CD248 | RAB11FIP4 | -0.533 | 5.12E-76 |
| CD248 | SLC10A6 | 0.579 | 8.32E-93 |
| CD248 | ABLIM3 | 0.514 | 6.12E-70 |
| CD248 | TMEM45A | 0.594 | 1.91E-98 |
| CD248 | SHMT2 | 0.516 | 1.48E-70 |
| CD248 | LINGO1 | 0.876 | 0 |
| CD248 | CARD16 | 0.537 | 1.58E-77 |
| CD248 | RAB33A | 0.546 | 1.70E-80 |
| CD248 | ANXA3 | -0.536 | 4.33E-77 |
| CD248 | RASAL3 | 0.542 | 4.34E-79 |
| CD248 | APLN | 0.794 | 8.51E-223 |
| CD248 | LGI4 | 0.651 | 2.42E-124 |
| CD248 | SLC43A3 | 0.697 | 7.40E-150 |
| CD248 | STAB1 | 0.701 | 2.85E-152 |
| CD248 | SLC6A1 | 0.675 | 8.73E-137 |
| CD248 | CHST15 | 0.623 | 3.08E-111 |
| CD248 | COL6A2 | 0.715 | 8.04E-161 |
| CD248 | IL21R | 0.588 | 4.61E-96 |
| CD248 | SLC2A3 | 0.74 | 2.14E-178 |
| CD248 | CD1D | 0.577 | 8.67E-92 |
| CD248 | SLC1A4 | 0.627 | 4.74E-113 |
| CD248 | SLC16A1 | 0.651 | 2.21E-124 |
| CD248 | GPR4 | 0.873 | 2.01578783503229e-321 |
| CD248 | NXPH4 | 0.583 | 4.24E-94 |
| CD248 | GNLY | 0.559 | 4.88E-85 |
| CD248 | TMEM178B | -0.505 | 3.22E-67 |
| CD248 | CXCL10 | 0.524 | 4.49E-73 |
| CD248 | IGFBP5 | 0.605 | 2.33E-103 |
| CD248 | UNC5B | 0.847 | 2.82E-282 |
| CD248 | ZNF395 | 0.657 | 1.35E-127 |
| CD248 | RUNX3 | 0.518 | 2.15E-71 |
| CD248 | ENPP3 | 0.55 | 8.23E-82 |
| CD248 | KISS1R | 0.569 | 1.13E-88 |
| CD248 | PGF | 0.772 | 3.84E-203 |
| CD248 | MAL | -0.576 | 1.96E-91 |
| CD248 | C3orf70 | 0.812 | 1.69E-241 |
| CD248 | CREB3L3 | 0.556 | 4.84E-84 |
| CD248 | EPHA3 | 0.745 | 1.12E-181 |
| CD248 | INHBB | 0.774 | 1.38E-204 |
| CD248 | HCLS1 | 0.55 | 4.37E-82 |
| CD248 | KCNK3 | 0.609 | 4.99E-105 |
| CD248 | EMCN | 0.589 | 1.16E-96 |
| CD248 | AFAP1L2 | 0.54 | 1.91E-78 |
| CD248 | COX4I2 | 0.786 | 1.70E-215 |
| CD248 | SPARCL1 | 0.672 | 3.79E-135 |
| CD248 | HECW2 | 0.722 | 1.19E-165 |
| CD248 | FATE1 | 0.691 | 4.41E-146 |
| CD248 | POSTN | 0.739 | 4.98E-177 |
| CD248 | NGF | 0.839 | 1.71E-272 |
| CD248 | GJC1 | 0.818 | 4.05E-247 |
| CD248 | SLC39A14 | 0.576 | 2.14E-91 |
| CD248 | PTHLH | 0.507 | 5.68E-68 |
| CD248 | RALGPS1 | -0.583 | 2.25E-94 |
| CD248 | PFKFB4 | 0.54 | 2.27E-78 |
| CD248 | FABP7 | 0.582 | 6.20E-94 |
| CD248 | PHKA2 | 0.643 | 1.76E-120 |
| CD248 | AGAP2 | 0.599 | 1.00E-100 |
| CD248 | CD248 | 1 | 0 |
| CD248 | C6orf223 | 0.673 | 5.47E-136 |
| CD248 | TPK1 | -0.507 | 6.45E-68 |
| CD248 | HEY1 | 0.627 | 4.50E-113 |
| CD248 | IL2RB | 0.594 | 1.39E-98 |
| CD248 | F2RL3 | 0.724 | 1.08E-166 |
| CD248 | MEIS2 | 0.532 | 8.97E-76 |
| CD248 | TMEM91 | 0.638 | 8.08E-118 |
| CD248 | CST7 | 0.505 | 2.39E-67 |
| CD248 | SNX33 | 0.528 | 1.15E-74 |
| CD248 | ARHGDIB | 0.73 | 1.25E-170 |
| CD248 | IGFBP3 | 0.66 | 4.33E-129 |
| CD248 | ADAMTS2 | 0.62 | 8.14E-110 |
| CD248 | PTPRE | 0.584 | 1.42E-94 |
| CD248 | PPP1R3B | 0.572 | 4.36E-90 |
| CD248 | CDK18 | 0.629 | 1.08E-113 |
| CD248 | DLK2 | 0.513 | 8.73E-70 |
| CD248 | LRRC20 | -0.565 | 1.67E-87 |
| CD248 | FAM189A1 | -0.504 | 4.89E-67 |
| CD248 | NPTX2 | 0.693 | 2.30E-147 |
| CD248 | COL14A1 | 0.572 | 8.09E-90 |
| CD248 | FABP6 | 0.522 | 1.50E-72 |
| CD248 | PDGFD | 0.526 | 8.53E-74 |
| CD248 | VASH1 | 0.639 | 3.01E-118 |
| CD248 | FGFBP2 | 0.585 | 3.69E-95 |
| CD248 | DDIT4 | 0.583 | 2.73E-94 |
| CD248 | CXCL9 | 0.576 | 1.21E-91 |
| CD248 | SPARC | 0.759 | 1.38E-192 |
| CD248 | GSTA2 | 0.508 | 4.03E-68 |
| CD248 | PLXDC1 | 0.868 | 4.69980729560209e-313 |
| CD248 | PALD1 | 0.643 | 2.58E-120 |
| CD248 | ADAMTS4 | 0.833 | 1.24E-264 |
| CD248 | MAP3K7CL | 0.564 | 6.40E-87 |
| CD248 | DLL4 | 0.835 | 4.61E-267 |
| CD248 | KCNJ2 | 0.607 | 4.07E-104 |
| CD248 | SERPINE1 | 0.693 | 5.57E-147 |
| CD248 | SLC2A1 | 0.652 | 6.40E-125 |
| CD248 | GNA14 | 0.603 | 2.27E-102 |
| CD248 | HAGHL | -0.515 | 3.18E-70 |
| CD248 | SCARB1 | 0.529 | 8.72E-75 |
| CD248 | CYP2J2 | 0.63 | 4.42E-114 |
| CD248 | RNF43 | -0.513 | 1.33E-69 |
| CD248 | SEMA5B | 0.639 | 1.07E-118 |
| CD248 | PARM1 | 0.502 | 2.41E-66 |
| CD248 | PLCXD2 | -0.568 | 1.26E-88 |
| CD248 | LCP2 | 0.6 | 6.37E-101 |
| CD248 | NDUFA4L2 | 0.8 | 9.76E-229 |
| CD248 | PPP1R18 | 0.589 | 2.22E-96 |
| CD248 | IDO1 | 0.594 | 1.59E-98 |
| CD248 | ESM1 | 0.757 | 6.41E-191 |
| CD248 | CXCR4 | 0.62 | 1.20E-109 |
| CD248 | GIT2 | 0.53 | 3.47E-75 |
| CD248 | PDE1B | 0.611 | 6.24E-106 |
| CD248 | LPCAT1 | 0.535 | 9.29E-77 |
| CD248 | MARVELD2 | -0.623 | 5.62E-111 |
| CD248 | ARHGAP15 | 0.62 | 6.77E-110 |
| CD248 | CBLC | -0.523 | 7.11E-73 |
| CD248 | COL21A1 | 0.652 | 5.30E-125 |
| CD248 | ILDR1 | -0.588 | 2.34E-96 |
| CD248 | RASSF2 | 0.603 | 3.47E-102 |
| CD248 | S1PR5 | 0.769 | 2.98E-200 |
