# Supplementary material for: The Diagnostic and Immunotherapeutic Value of CD248 in Renal Cell Carcinoma
Source: Front Oncol. 2021 Mar 12;11:644612. doi: 10.3389/fonc.2021.644612 (PMC8006336; doi:10.3389/fonc.2021.644612)
Supplement: Supplementary file 3 [file Table_3.DOCX]

**Supporting data 3** CD248-correlated PDEmiRNA

| **Gene 1** | **Gene 2** | **Pearson correlation coefficient** | ***P* value** |
| --- | --- | --- | --- |
| CD248 | hsa-miR-503-5p | 0.785 | 3.15E-182 |
| CD248 | hsa-miR-30d-5p | 0.783 | 2.17E-180 |
| CD248 | hsa-miR-25-5p | 0.782 | 1.66E-179 |
| CD248 | hsa-miR-655-3p | 0.78 | 3.26E-178 |
| CD248 | hsa-miR-517c-3p | 0.777 | 2.36E-176 |
| CD248 | hsa-miR-616-5p | 0.776 | 2.23E-175 |
| CD248 | hsa-miR-16-5p | 0.774 | 1.43E-173 |
| CD248 | hsa-miR-196a-5p | 0.771 | 9.81E-172 |
| CD248 | hsa-miR-20b-5p | 0.769 | 2.29E-170 |
| CD248 | hsa-miR-23a-5p | 0.769 | 1.96E-170 |
| CD248 | hsa-miR-211-5p | 0.766 | 2.07E-168 |
| CD248 | hsa-miR-17-5p | 0.765 | 1.61E-167 |
| CD248 | hsa-miR-382-5p | 0.761 | 6.39E-165 |
| CD248 | hsa-miR-411-5p | 0.759 | 2.59E-163 |
| CD248 | hsa-miR-493-5p | 0.759 | 2.08E-163 |
| CD248 | hsa-miR-150-5p | 0.756 | 1.87E-161 |
| CD248 | hsa-miR-34a-5p | 0.756 | 3.03E-161 |
| CD248 | hsa-miR-632 | 0.751 | 4.64E-158 |
| CD248 | hsa-miR-374a-5p | 0.749 | 6.58E-157 |
| CD248 | hsa-miR-555 | 0.739 | 2.64E-150 |
| CD248 | hsa-miR-615-3p | 0.721 | 3.41E-140 |
| CD248 | hsa-miR-21-5p | 0.7 | 1.56E-128 |
| CD248 | hsa-miR-629-3p | 0.692 | 1.56E-124 |
| CD248 | hsa-miR-199a-3p | 0.668 | 4.53E-113 |
| CD248 | hsa-miR-454-3p | 0.664 | 1.98E-111 |
| CD248 | hsa-miR-602 | 0.651 | 1.68E-105 |
| CD248 | hsa-miR-425-5p | 0.629 | 9.85E-97 |
| CD248 | hsa-miR-377-3p | 0.62 | 3.31E-93 |
| CD248 | hsa-miR-488-5p | 0.605 | 1.47E-87 |
| CD248 | hsa-miR-221-3p | 0.598 | 2.42E-85 |
| CD248 | hsa-miR-126-5p | 0.579 | 9.00E-79 |
| CD248 | hsa-miR-376b-3p | 0.552 | 2.31E-70 |
| CD248 | hsa-miR-505-3p | 0.531 | 3.22E-64 |
| CD248 | hsa-miR-519e-3p | 0.52 | 3.85E-61 |
| CD248 | hsa-miR-410-3p | 0.501 | 2.44E-56 |
| CD248 | hsa-miR-181d-5p | -0.573 | 1.13E-76 |
| CD248 | hsa-miR-21-3p | -0.582 | 6.81E-80 |
| CD248 | hsa-miR-216a-5p | -0.618 | 1.72E-92 |
| CD248 | hsa-miR-10b-5p | -0.639 | 1.28E-100 |
| CD248 | hsa-miR-329-3p | -0.668 | 3.02E-113 |
| CD248 | hsa-miR-516b-5p | -0.668 | 2.97E-113 |
| CD248 | hsa-miR-373-3p | -0.71 | 4.26E-134 |
| CD248 | hsa-miR-433-3p | -0.711 | 1.20E-134 |
| CD248 | hsa-miR-203a-3p | -0.715 | 1.02E-136 |
| CD248 | hsa-miR-509-3p | -0.721 | 1.10E-139 |
| CD248 | hsa-miR-519a-3p | -0.724 | 6.71E-142 |
| CD248 | hsa-miR-549a | -0.738 | 8.89E-150 |
| CD248 | hsa-miR-17-3p | -0.744 | 1.45E-153 |
| CD248 | hsa-miR-432-5p | -0.746 | 4.56E-155 |
| CD248 | hsa-miR-25-3p | -0.747 | 1.59E-155 |
| CD248 | hsa-miR-193b-3p | -0.75 | 3.64E-157 |
| CD248 | hsa-miR-214-3p | -0.762 | 1.72E-165 |
| CD248 | hsa-miR-215-5p | -0.764 | 5.15E-167 |
| CD248 | hsa-miR-218-5p | -0.787 | 1.01E-183 |
